# Hospital and Institutional News

**Published:** 1914-02-07

**Authors:** 


### HOSPITAL AND INSTITUTIONAL NEWS.

### AN INSTITUTE OF PATHOLOGY AT THE MIDDLESEX HOSPITAL.

The board of the Middlesex Hospital are advertising for a whole-time director of the new Institute of Pathology, at a salary of £800 per annum. Hitherto the clinical and bacteriological laboratories and the pathological departments have been kept separate; but now that the new Institute of Pathology is established, all research and teaching in pathology and hygiene will be centralised and placed under the control of one man with a suitable staff of assistants. The Institute will be quite separate from the Cancer Charity and its laboratories. We learn that the board require their director to be under forty years of age; to undertake no private work during his official hours, except that he may hold one examining post at a time; and to retire on arriving at the age of sixty. The scheme seems an excellent one, and the conditions should attract plenty of first-rate candidates. We congratulate the Middlesex Hospital board on their courage in breaking fresh ground in this way, and in deciding to spend money freely in order to get the best kind of officer.

### MENTAL HOSPITALS AND EARLY CASES.

The present condition of the lunacy laws and the need for reforming them, not only in the interests of science but also of the early case, led Dr. Edwin Goodall, medical superintendent of Cardiff Mental Hospital, to discuss in a local paper the present factors in this problem. It is an extraordinary fact, he noted, that nowhere in this country can a poor person obtain indoor treatment at a public mental hospital, except at the price of his personal liberty. This he stigmatised as a grave injustice, which had been brought about by neglect of the fact that a sick man as well as a citizen had to be dealt with. As a result of the lawyers having it their own way, through the medical profession standing aloof, excessive segregation was enforced, which militated against recovery. Dr. Goodall wisely pointed out that this had led also to the partial segregation of the mental hospital staffs, with results that we all know and regret for the sake of the service. The remedy, which he prescribed in the *Western Mail*, was to alter the law so that borderland cases may be able to attend the out- and in-patient departments of the ordinary hospital. The West Riding Mental Hospital possesses an out-patient department, but patients object to attending one associated with an "asylum," and the argument is for separate clinics without that popular disadvantage.

### THE PSYCHIATRIC CLINIC AND THE VOLUNTARY SYSTEM.

To enable patients to be received and detained without certificates, which is what Dr. Goodall advocates, the physician in charge must have more freedom than is at present allowed. The clinic would serve also as a centre of research and training in psychiatry, as it does now in the Colonies and in America, and would, partly by this means and partly by opening "horizons round" to the younger members of the mental service, make that service more attractive to suitable candidates who would be retained, which is often not the case as things are. In advocating the adoption of the psychiatric clinic in connection with the medical schools, Dr. Goodall said that the great difficulty in the way was the voluntary principle, which made the hospitals, as a rule, suffer from lack of funds. That, no doubt, is the inevitable impression of a man in a publicly supported institution, but he forgets that the chief difference really is that in one case the public hears nothing about how the money is raised, while in the other the whole policy is to inform them of everything. But the constant talk about money and appeals must not be confused with chronic impecuniosity. Nothing really requiring to be done is too expensive for the voluntary system. We therefore welcome Dr. Goodall's restatement of the case for the psychiatric clinic, because we believe that it is one in which public opinion has the greatest possible need to be coached. Dr. Goodall's concluding warning must not be forgotten. If the new Maudsley Mental Hospital, he said, is to be worked under the existing lunacy laws it will merely be a glorified "asylum," and that is the danger which he foresaw. The Medico-Legal Society's recent resolution that "the early treatment of incipient insanity without certification will be advantageous" should help to focus public attention on the nature of the new legislation required.

### LEANCHOIL HOSPITAL AND LORD STRATHCONA.

Sir John Macpherson Grant, of Ballindalloch, as president of Forres Leanchoil Hospital, Morayshire, made a striking reference to the institution's debt to the late Lord Strathcona. Forres, he said, was his home, and they all remembered the old house by the bridge. If they required any monument to his memory they had only to say in Roman fashion "Look about you." They all knew his record and also that the hospital owed a great deal, if not everything, to him. Leanchoil Hospital, which was founded in 1892, and of which Mr. H. Saxon Snell was the architect, is an institution of twenty beds, and also for the past six years has possessed an isolation ward for infectious diseases. Lord Strathcona was always regarded affectionately as the benefactor of the hospital, which, from Forres being his family's place—his father and mother are buried in the churchyard—has always been associated closely with his interests. It is, therefore, not surprising to see that his will contains a bequest of £10,000 to this institution.

### A HOSPITAL AND PARTY POLITICS.

We note this week a curious provision in a will by which a voluntary hospital becomes a beneficiary if and when the minister of a certain Methodist chapel indulges in any form of party politics. Mr. Edward Trounson, of Redruth, merchant, has left £1,000 to the West Cornwall Miners' and Women's Hospital, Redruth, and 200 £5 "A" Preference shares in S. and T. Trounson, Ltd., or the proceeds of the sale thereof, to the trustees of the United Methodist Chapel, Fore Street, Redruth, upon trust for the better payment of the pastor regularly officiating in that chapel, but only so long as such minister shall not take part in public party politics, whether on the platform or in committee rooms or elsewhere, and if such minister shall so take part, the income shall, during such time as he shall be connected with that chapel, be applied for the benefit of the West Cornwall Miners' and Women's Hospital. Hospital managers nowadays are apt to regard the voluntary system so naturally as the universal residuary legatee of testators, that they forget the tremendous amount of hard work which their elders have accomplished to achieve such a wonderful measure of public confidence.

### STAFF CHANGES AT CLAYBURY HOSPITAL.

THE number of changes which has been made recently in the medical staff at Claybury Mental Hospital suggests that the Mental Hospitals Committee of the London County Council is still finding difficulty in filling up the ranks of its assistant medical officers, despite the improvements which have recently been made in the conditions of service. Mr. O. H. Bowen having relinquished his appointment as fourth assistant medical officer, Mr. E. G. T. Poynder and Mr. W. Moodie have been promoted fourth and fifth assistant medical officers respectively. Mr. D. W. Rintoul was appointed sixth assistant, but has since resigned and left the service, and Mr. Poynder also has resigned his post to take up another appointment in the Egyptian Service.

### A DISCUSSION ON SANATORIA CONSTRUCTION.

Now that the cost and construction of sanatoria under the Insurance Act is exercising the minds of so many persons and local authorities considerable interest attaches to the meeting that will be held at the Royal Sanitary Institute, 90 Buckingham Palace Road, S.W., on Tuesday next, the 10th inst., when at 7.30 P.M. Mr. Edwin T. Hall, F.R.I.B.A., will read a paper on "The King Edward VII. Welsh National Memorial Sanatorium, Pant-y-Wal, South Wales." Mr. Hall is, of course, the architect to whom the building was entrusted, and a discussion will follow on the sanatoria, their design and expense, required under the Insurance Act. Mr. David Davies, M.P., will preside, and if good use be made of the occasion a fruitful exchange of views ought to take place.

### LORD STRATHCONA'S HOSPITAL BEQUESTS.

IT is interesting to note that the late Lord Strathcona, who has settled his Scottish estates and half a million on the heirs succeeding to the title, has bequeathed a sum of £475,000 to public purposes and charities. And of this, hospitals, especially Canadian hospitals, receive an important share. The Royal Victoria Hospital, Montreal, receives the substantial sum of £100,000, and for the rest we note that Queen Alexandra Extension Home and Hospital for Incurables, Streatham, the National Hospital for Paralysed and Epileptics, University College Hospital, and the Middlesex Hospital benefit to the extent of £2,000 apiece. Under another heading we record his bequest of £10,000 to the Leanchoil Hospital, and his early association with that place. We may be sure that so long as the present tendency to support the voluntary system persists at its high degree of vigour, there will not be the smallest temptation for public authorities to pay the least heed to those scaremongers who, depressed by some local difficulty, raise their little pipe in favour of what they call "municipalisation."

### THE REGISTRATION OF STILL-BIRTHS.

WE have often pointed out the curious discrepancy in the statistics compulsorily registered in Great Britain—namely, that there is no compulsory registration of still-births in this country. In the state of confusion in the public mind which now exists over the whole problems of the birth-rate it is extremely important that the factor of still-births and the proportion that these bear to the general rate should not be neglected. Dr. Reginald Dudfield, the medical officer of health for Paddington, has therefore done something useful in again attracting attention to this anomalous omission by publishing certain figures which he has collected bearing upon the subject. By means of a system of index numbers he shows the proportion which infantile mortality plays in the total death-rate. What is needed is the classification of the still-births themselves, so that data may be available for fixing the responsibility in due proportion upon each of the predisposing causes, such as congenital defect, the nature of maternal occupation, and so forth. As a matter of fact the science of statistics is almost the youngest of the social sciences, even in its most rudimentary form, the Census—it is little more than fifty years old—and weather reports have not been in the eternal nature of things so long as newspaper readers imagine. But to have a register of births which does not take any account of still-births is a gratuitous omission that cannot be allowed to endure.

### QUEEN ALEXANDRA AND ST. KATHARINE'S HOSPITAL.

AN important announcement with regard to the future of St. Katharine's Hospital was made at Tuesday's meeting of the London County Council. Fifteen months ago the Council had the future of the hospital under consideration, but no action was taken, as it was understood that Queen Alexandra was considering the whole question, to which attention has been drawn on more than one occasion in THE HOSPITAL. At Tuesday's meeting Mr. E. L. Meinertzhagen announced that new rules relating to St. Katharine's Hospital had been prepared by the Lord Chancellor under authority given by Queen Alexandra, and had been communicated to the Council. The Local Government Committee would report to the Council later on the subject, and at that stage it would be sufficient for him to say that the primary object of the new rules was to restore to the poor of the East of London the benefits of the foundation by establishing near the site of the ancient foundation a college for the provision of a body of qualified resident women health workers, and the training for such work of women students. There seems to be every prospect, therefore, that the East of London will regain the benefits which it enjoyed before the hospital was removed early in the nineteenth century from its original site near the Tower of London to Regent's Park.

### ST. GEORGE'S HOSPITAL SPECIAL COURT.

We desire again to make clear that the date of the Special Court of Governors of St. George's Hospital has been fixed for Thursday, February 12, 1914, not the 11th, as previously announced. We are requested by Mr. Stuart de la Rue to state that the large number of letters received in response to the announcement in The Hospital of January 17 has made it impossible to deal with them all individually, and that he desires to thank those who have communicated with him, and will do his utmost to keep them informed of the course of events, especially should they be abroad.

### SALARIES OF TUBERCULOSIS OFFICERS.

The great demand made on the services of specialists, who have made particular study of tuberculosis, has led medical men, with a training that has equipped them to cope with the disease, to find that they can command salaries that previously were out of their reach, and, as the number of tuberculosis dispensaries increase, there comes a rise in the scale of pay. This is being felt by the authorities, who before the Insurance Act came into operation or in its earlier stages engaged tuberculosis specialists. A short experience in a municipal dispensary qualifies the tuberculosis officer to look out for the more remunerative posts that have been created during the last twelve months, or entitles him to ask for an increase of salary. In many cases the authority under whom he serves has no option except to grant the extra remuneration asked. Such a case has occurred in Lambeth. Dr. S. N. Gilbraith, the assistant medical tuberculosis officer, was appointed in February last year at a salary of £300 a year, which was stated to be a "commencing" one. At the close of twelve months' service he applied for an increase, and the council has decided to give him an additional £50 a year. Asked whether this was the maximum salary attached to the office, Alderman Gibbs acknowledged that no maximum had been fixed. As a councillor remarked, the borough had been saddled with an expenditure which they never thought of when the Act came into force. The increased expenditure by Metropolitan borough councils, due to the Insurance Act, is not confined to Lambeth. In Wandsworth the council has been compelled to accept £100 from the Insurance Committee for the County of London in settlement of their account for the treatment of insured persons at the municipal dispensaries, though the council well recognised that the sum did not reimburse them for the money they had expended on the people whose ailments the Insurance Act was supposed to cover. It was contended that it was better to accept this modified sum rather than to attempt to get more, with a possibility of getting nothing at all. Some borough councils, who have been treating insured persons at their dispensaries, have received nothing. Thus it is contemplated by some that the Act will be responsible for at least a penny increase in the rates in March next. Yet if the tuberculosis dispensary is to do all that its advocates claim for it, it is essential that the right type of man be attracted to and retained in the responsible post.

### THE SINGLE PAVILION: A FORECAST.

Taking advantage of an appeal for balconies made in the report presented by Mr. F. W. Archer, the secretary of the Birkenhead and Wirral Children's Hospital, last week, Dr. Francis Johnston forecasts the future of hospitals as single-storeyed pavilions in the open country. He said that the several-storeyed building of the town was becoming a thing of the past, and that hospitals would consist of a number of pavilions on country estates, like that at Arrowe Hall, near Woodchurch. In country hospitals for children he particularly advocated the providing for a large number, such as 1,500 or 2,000, so that weakly, anæmic children might remain for long periods, and carry out their education while under the hospital's care. Everyone knows the Liverpool country hospital at Heswall, and has watched, for this class of institution in particular, the passing of the closed ward, and the way in which a cottage block plan, as at Carshalton, allows by the addition of balconies practically double the accommodation afforded by the buildings themselves. We are at the beginning, rather than the end, of hospital design, and consequently many hesitate to praise the great reconstructions on the old plan that some of our cities are undertaking in the centre of their business activity.

### NEW POOR-LAW HOSPITAL AT NEWCASTLE.

The Lord Mayor of Newcastle, Councillor Johnstone Wallace, formally opened, last week, the first instalment of a great scheme for a thousand beds in connection with the Newcastle Workhouse. It provides accommodation for 154 patients in wards, the largest of which contains twenty-eight beds in a three-storeyed building. On the ground and first floors there are two large wards, each 97 feet long by 24 wide, with four single-bed wards, day-rooms, linen, bath, stores, and sterilising-room and two ward-kitchens. The first floor is built on the same plan, but the second floor is devoted to incipient phthisical cases, and consists of a number of small wards and cubicles, which open off a glass corridor in such a way that the free circulation of air is unhindered. There is also a flat, partly open and partly covered in, allowing beds to be out of doors in all weathers. Mr. Edwin Bowman, of Newcastle, is the architect. Mr. Lonsdale, chairman of the Guardians, stated that the entire scheme referred to would comprise four similar blocks, together with one for administration purposes, and that it would be proceeded with as occasion arose.

### MR. HOLLAND SUCCEEDS TO THE PEERAGE.

By the death of Lord Knutsford at the advanced age of eighty-eight, Mr. Sydney Holland, the chairman of the London Hospital, succeeds to the peerage. Lord Knutsford's career was mainly legal and political, and he will be remembered as Colonial Secretary, to which office he was appointed in 1887, and which he held for five years. From the hospital point of view it may be noticed that he was a Sub-Prior of the Order of St. John, in the ambulance work of which he always took a keen interest. Mr. Holland's elevation to the peerage has come, from the public point of view, at a fitting time, since it coincides with the success of the London Hospital's latest quinquennial appeal, on which we remarked only recently. The charitable public, and indeed many not so benevolently inclined, will miss the well-known signature which for so long has accompanied the active personal appeals which have emanated under his chairmanship from the London Hospital. But the good work, we may be sure, will continue, and it will be an advantage to the whole voluntary hospital system to have, as it will now, an equipped spokesman for its interests in the House of Lords in the person of the second Lord Knutsford.

### ALLEGED NEGLIGENCE OF AN ANÆSTHETIST.

It is worth while noting the remarks made by a judge last week in giving a verdict for the defendants in an action brought by an infant scholar who claimed damages for personal injury caused by the alleged negligent administration of an anæsthetic. The anæsthetist and the nurses who were present at the operation having given evidence, and the jury finding that the anæsthetist had not been guilty of negligence, Mr. Justice Lush, in giving judgment, said that in his opinion a contrary verdict would have imposed no liability on the defendants, as their duty to the plaintiff was discharged when they engaged competent professional men to perform the operation. We take it that the County Council and other bodies will note this expression of opinion carefully, and that its publication, if remembered, will do much to discourage actions like the above from being brought.

### NEW PREMISES AT NORWICH.

The Norfolk and Norwich Eye Infirmary held its meeting for the first time last week in the new premises since its removal from Pottergate Street. In 1912 an arrangement was made with the Norfolk and Norwich Hospital to alter and equip Grove House as a new Eye Infirmary. The Corporation has agreed to purchase the old premises, for the new in-patient department was opened by the Duke of Norfolk in July last, and the new out-patient department, the gift of Mr. Arthur Samuel, then Lord Mayor, was ready for work in December. The old buildings were certainly out of the way, and the new institution, besides being better situated, is, as Mr. S. Cozens Hardy put it, in closer touch with the Norfolk and Norwich Hospital.

### FACTORY LECTURES ON VENEREAL DISEASE.

The eleventh meeting of the Royal Commission on Venereal Disease was remarkable for a suggestion which was made by Lieutenant-Colonel Gibbard, R.A.M.C., head of the Rochester Row Royal Military Hospital, with reference to the education of the civil population. He stated that the experience of the military hospital of which he was in charge had shown the value of lectures, and argued from this that it would be an advantage for a course of lectures to be given in all factories by selected medical men and women, according to the sex of the employees, and that these might be illustrated with kinemacolour photographs. The lectures, he added, should insist especially upon the importance of seeking medical advice on the earliest suspicion of contagion, and of eschewing chemists and quacks. We shall be curious to learn how this suggestion is greeted by factory owners and their employees. It is at any rate true, in spite of the denials that are often made so thoughtlessly, that lectures are really extremely popular forms of recreation, in the best sense of that word, and that lecture-halls are often more easily filled than theatres and places of competitive amusement.

### RED CROSS AEROPLANES.

It is gratifying to learn through Lieutenant-Colonel W. C. Beevor, Staff Officer of the North Midland Division, Staffordshire Territorial Force, that the War Office intends to allow the use of an aeroplane to the British Red Cross Society during the coming summer to demonstrate its value as a form of transport for the wounded. Our readers will remember the illustrated article which we published on August 23 last year by Lieutenant-Colonel J. D. F. Donegan on "The Probable Uses of Aerial Transport to the Medical Profession," in which the writer anticipated that airships would replace ambulance trains, and saw "no reason why aerial transport should not be used for the removal of the injured." Reference was also made to the experiments which had been tried with the late Colonel Cody's 100 horse-power machine. Colonel Donegan, in conclusion, remarked that this ill-fated airman had intended to devote his machine to ambulance work, even to try to rig up contrivances to carry helpless lying-down cases on the wings. This summer, we hope, will see the success of some of these or similar projected experiments, which Lieutenant-Colonel Beevor expects will be undertaken in the Victoria Park, Leicester. It will not be a little curious if it is the aeroplane which leads the War Office to increase the somewhat grudging attention which it has given to Red Cross work.

### STAFF CHANGES AT BRENTFORD INFIRMARY.

At the last meeting of the Brentford Guardians the general purposes committee reported that it had received the resignation of Dr. O. W. D. Steel, second assistant to the medical superintendent of the infirmary, and that it had authorised the appointment of a *locum tenens*. The Guardians decided that the salary to be assigned to the office in future should be £150, rising by annual increments of £10 to £170, together with the usual residential emoluments, valued for superannuation purposes at £75 per annum. At the same meeting Dr. James Rae, M.B., Ch.B., M.D., of the Selly Oak Infirmary, Birmingham, was appointed as first assistant to the medical superintendent of the infirmary and medical officer of the workhouse and schools. He is the author of "The Deaths of English Kings."

### THE MACHINERY OF THE COUNTY COUNCIL.

A committee of the London County Council, including Mr. Cyril Cobb, the chairman, and Lieut. Sladen, chief of the Fire Brigade, inspected three types of motor ambulance at Spring Gardens last week. One of the three was that subsidised by the Council, and owned by the Hampstead General and North-West London Hospital. A dummy patient—that is to say, a healthy man weighing some twelve stone—was used to test the comfort of each vehicle, the largest of which was a Daimler type, costing some £1,000, but which some believed to be, in spite of its great virtues, almost too big for use in thickly crowded streets. The Bishopsgate Ambulance Station also received a visit of inspection, and a speed test was undertaken at Guildhall Yard, from which a call was sent to the Bishopsgate Station. In four and a half minutes the ambulance arrived, a time noteworthy from the fact that the heaviness of the traffic in this district happened also to be combined with greasy roads. The experiences of the day were, no doubt, instructive, but the fact remains that it is not the machinery of the various types of ambulance which needs speeding up, but the machinery of the London County Council.

### THE HOSPITAL FOR POOR JEWS AT JERUSALEM.

A correspondent sends us the following particulars about the Medical Mission at Jerusalem:—"The new hospital of the Jerusalem Medical Mission in connection with the Society for Promoting Christianity amongst the Jews, which was opened in 1897 (though the Medical Mission itself was founded in 1824 and numbers among the names of its medical men those of Drs. Dalton, Macgowan, Nichols, Sandford, Sims, Simpson, Atkinson, Chaplin, D'Erf Wheeler, and many others, and a hospital has been in existence from 1844), is built on a semi-circular plan and upon the pavilion system. The centre of the semi-circular block contains the administrative offices, theatre, and nurses' quarters, at the extreme east of the semi-circle is the out-patient department, and at the extreme west the house of the senior medical officer, Dr. Masterman. There are some six wards—the 'Norfolk and Norwich' and the 'Cadbury Memorial' wards for men, the 'Chaplin' and 'Meath' wards for women, and two 'Axford Memorial' wards for children. There are in all 69 beds—19 for men, 26 for women, and 24 for children—available for poor Jews, with the addition of three single-bedded wards for travellers. The hospital staff consists of two medical missionaries, six English nurses, two dispensers, and some native assistants, who are taken into the hospital to be trained as probationers, and when it is stated that there is usually one nurse on furlough, one on night duty, and one working on a certain district it will be seen that the work is considerable. There are two kitchens situated in the administrative block, one for the preparation of 'Kosher' food, which is bought, cooked, and served only by Jews, the other for the food for the staffs, etc.—that is the Gentile kitchen—at the extreme rear of the central block is a small isolation block. The work of the Medical Mission lies not only within the hospital, but home visits are paid by the staff and dispensaries have been opened both in the city and at Siloam. The opposition of the Rabbis is naturally a great hindrance, but the gratitude of the people is very real and there is a great opportunity for work."

### A COTTAGE HOSPITAL'S LITIGATION.

It is, luckily, not often that a cottage hospital is involved in litigation, and the experience of the Watton Victoria Cottage Hospital during the past year well bears this out. The hospital drainage was the cause of the trouble, and in the end an injunction was applied for, which, on the advice of their solicitors, the hospital decided to oppose. The result was unfortunate, as they lost their case and were cast in costs, which involved a considerable outlay, apart altogether from the expense of laying a new system of drainage which was rendered necessary. The success which this is proving is but a small consolation to the authorities, and at the same time Mr. Charles Robinson, the secretary, resigned after a connection of fourteen years with the hospital, which, in fact, he assisted to build. He also took an active part in raising the funds which were required by the children's ward and the operation room. His successor in the office of secretary is Mr. F. W. Stimpson, and we trust that the New Year will be one of less anxiety to the management.

### THE NEW GLASGOW CHILDREN'S HOSPITAL

The new institution, to be occupied by the Royal Hospital for Sick Children, Glasgow, is nearing completion at Yorkhill, and it is expected to be ready to start work in the course of the coming summer. It will be remembered that an appeal for £30,000 has been issued, and it is satisfactory to add that, according to the latest report, about half this sum has been subscribed or promised. Sir

John Ure Primrose referred at a recent meeting to the supplementary work done at the country branch, and Dr. Kennedy Dalziel pointed out that critics of the new site in the centre of Glasgow forgot that the hospital was not intended for the prolonged treatment of cases, and also that it must be near the University, because of the teaching work, which was one of its great functions. As to the financial side of the matter, it has been decided to issue an appeal for the rest of the money, and one speaker expressed the belief that the Corporation would come to their assistance, it having subscribed £3,000 on a former occasion. Indeed, Mr. C. K. Aitken remarked that the directors had no anxiety about the remaining capital sum required, but that the cost of maintenance might prove a difficulty. The old institution had always paid its way, but, instead of the cost being £8,000, as at present, probably £20,000 would be needed for the new institution. The opening will be looked forward to by all who have watched the progress of the scheme, and, knowing Glasgow's generous support of voluntary institutions, there can be little doubt that the money will be quickly raised.

### A NOTABLE COTTAGE HOSPITAL TREASURER.

We are glad to note that the presentation made to Major R. C. Tucker at the time of his resignation of the office of honorary treasurer to the Ashburton and Buckfastleigh Cottage Hospital a little while ago has been followed by a formal resolution as an historical record of his services. For, indeed, his connection with the institution has been remarkable, since he has held the office of treasurer since the hospital's foundation, that is to say, for a period of thirty-six years. All who have watched the growth of the cottage hospital, and indeed the emergence of these institutions into a class to which the name "cottage" cannot justly apply, will recall the opposition with which they were often met at their commencement. No doubt Major Tucker has recollections of that time which would surprise the existing generation of subscribers who take the cottage hospital as a matter of course, and regard it as an honourable and invaluable part of provincial organisation, the use of which it would be impossible to question.

### THE GROWTH OF THE GERMAN HOSPITAL.

The average institutional worker does not know as much as he ought about the various foreign hospitals of the Metropolis, which come mainly into public notice when the official representative of the country whose "colony" they treat pays a visit here, of which occasion is taken for an inspection of the institution. The past year at the German Hospital, Dalston, has been an eventful one, for it saw the completion of the new block, consisting of two small wards and staff quarters, on the south side of the institution, by which twelve additional beds were added to the accommodation, which now comprises 154 beds. The *x*-ray department has been reconstructed, and the new laundry and disinfecting room completed. In the meantime, we note that a new out-patient department is engaging the attention of the committee, which is also considering the question of a new mortuary, and if the plans now under review are adopted a sum of £20,000 will be appealed for to carry them out. It is only fair to remind our fellow-countrymen that of the 2,358 in-patients 537 were English, and that should be a good argument for the subscriptions of those who might otherwise fancy that a "German" hospital had little claim upon their pockets. It is interesting also to add that 150 private patients were received—an entry generally that is likely to grow more common now that what may be called the revolt against the nursing home has set in.

### PUBLIC PHARMACISTS' ASSOCIATION'S NEW CHAIRMAN.

Mr. Jas. Hassall France, M.P.S., who now becomes the chairman of the Public Pharmacists' Association, is well known to London institution pharmacists, having acted for several years as honorary secretary to the Association. He has been a public servant for many years, and now holds the post of pharmacist at the City of Westminster Union Dispensary in Buckingham Palace Road, S.W. His pharmaceutical associations are extensive, his father being a prominent chemist in Rotherham, Yorks, while he himself has held important engagements in Birmingham, Brighton, and the West End. Mr. France has become a strong adherent to motor-cycling as a hobby, and hopes by this means to be able to see many public pharmacists during his term of office. The policy the Association is now strongly advocating is the adoption by the Local Government Board of the title "pharmacist," thus bringing this branch into line with the Admiralty and the Home Office, which have adopted it for the Navy and Prison Service.

### THIS WEEK'S DRUG MARKET.

The improvement in business noted last week has been maintained, and the prospects appear to be good. Menthol has attracted less attention, but the recent advance in price was probably justified by the statistical position of the article, and will probably be maintained for the present; but the menthol market often surprises those who know it best. Opium is practically unchanged, but there is a possibility of rather lower prices; morphine and codeine are unaltered in price. Little definite news of the cod fishing has come from Norway as yet, and buyers are still holding off; reports so far to hand are conflicting. There has been a small reduction in the price of cocaine, and the quotation is now sufficiently low to tempt buyers to replenish their stocks. Wool fat (both hydrous and anhydrous) has advanced in price. Cantharides are again dearer. Chamomiles are somewhat lower in price. Indian hemp is dearer, and still higher prices are not improbable. Castor oil is slightly lower in price. Thymol is again rather dearer. Cream of tartar has an upward tendency, but citric acid tends slightly downwards.

